# The Translation Initiation Factor 1A (*TheIF1A*) from *Tamarix hispida* Is Regulated by a Dof Transcription Factor and Increased Abiotic Stress Tolerance

**DOI:** 10.3389/fpls.2017.00513

**Published:** 2017-04-07

**Authors:** Guiyan Yang, Lili Yu, Yucheng Wang, Chao Wang, Caiqiu Gao

**Affiliations:** State Key Laboratory of Tree Genetics and Breeding, Northeast Forestry UniversityHarbin, China

**Keywords:** eIF1A, expression, abiotic stress, promoter, *Tamarix hispida*

## Abstract

Eukaryotic translation initiation factor 1A (*eIF1A*) functions as an mRNA scanner and AUG initiation codon locator. However, few studies have clarified the role of *eIF1A* in abiotic stress. In this study, we cloned *eIF1A* (*TheIF1A*) from *Tamarix hispida* and found its expression to be induced by NaCl and polyethylene glycol (PEG) in roots, stems, and leaves. Compared to control, *TheIF1A* root expression was increased 187.63-fold when exposed to NaCl for 6 h, suggesting a potential abiotic stress response for this gene. Furthermore, transgenic tobacco plants overexpressing *TheIF1A* exhibited enhanced seed germination and a higher total chlorophyll content under salt and mannitol stresses. Increased superoxide dismutase, peroxidase, glutathione transferase and glutathione peroxidase activities, as well as decreased electrolyte leakage rates and malondialdehyde contents, were observed in *TheIF1A*-transgenic tobacco and *T. hispida* seedlings under salt and mannitol stresses. Histochemical staining suggested that *TheIF1A* improves reactive oxygen species (ROS) scavenging in plants. Moreover, *TheIF1A* may regulate expression of stress-related genes, including *TOBLTP*, *GST*, *MnSOD*, *NtMPK9*, *poxN1*, and *CDPK15*. Moreover, a 1352-bp promoter fragment of *TheIF1A* was isolated, and *cis*-elements were identified. Yeast one-hybrid assays showed that ThDof can specifically bind to the Dof motif present in the promoter. In addition, *ThDof* showed expression patterns similar to those of *TheIF1A* under NaCl and PEG stresses. These findings suggest the potential mechanism and physiological roles of *TheIF1A*. *ThDof* may be an upstream regulator of *TheIF1A*, and *TheIF1A* may function as a stress response regulator to improve plant salt and osmotic stress tolerance via regulation of associated enzymes and ROS scavenging, thereby reducing cell damage under stress conditions.

## Introduction

Eukaryotic initiation factors (eIFs), including six different families with various functions, namely, eIF1, eIF2, eIF3, eIF4, eIF5 and eIF6, are involved in translation initiation in eukaryotes (Howarth, 1991; Pyronnet and Sonenberg, 2001; Dong and Zhang, 2006). In addition, eIFs are involved in aspects of stress regulation. For example, strong heat shock in *Saccharomyces cerevisiae* induces assembly of stress granules containing eIF3 that is independent of eIF2α phosphorylation (Groušl et al., 2009). *TaeIF3g* is expressed in response to mild osmotic stress, an effect that differed in the grains of two wheat cultivars (Singh et al., 2007). *eIF4E* isoform 2 is a salt-related factor in *Schizosaccharomyces pombe* (Ptushkina et al., 2004), whereas *eIF5A* was initially identified as a factor involved in the formation of the first peptide bond in rabbit reticulocytes (Li et al., 2004; Costa-Neto et al., 2006). *eIF5A* was subsequently identified as a factor important for plant adaptation to changing environmental conditions by increasing protein synthesis and enhancing ROS scavenging. This latter aspect is related to increased SOD and POD activities as well as the prevention of chlorophyll loss and membrane damage (Wang et al., 2012).

The translation initiation factor *eIF1A* is necessary for directing the 43S preinitiation complex from the 5′ end of an mRNA to the initiation codon in a process referred to as ‘scanning.’ In humans, *eIF1A* contains an oligonucleotide-binding (OB) fold and binds to single-stranded RNA oligonucleotides in a site-specific but non-sequence-specific manner, suggesting mRNA interaction rather than specific rRNA or tRNA binding (Battiste et al., 2000). Translation initiation proceeds via an initiator tRNA and the start codon of an mRNA positioned in the ribosomal P site. In eukaryotes, one of the first steps involves the binding of *eIF1* and *eIF1A* to the 40S ribosomal subunit *eIF1* and the *eIF1A* promoter in an open, scanning-competent preinitiation complex that closes following start codon recognition (Passmore et al., 2007). Previous studies have demonstrated that *eIF1A* participates in plant developmental and stress regulation. For instance, overexpression of sugar beet *eIF1A* specifically increased sodium and lithium salt tolerance in yeast and Arabidopsis, which suggests that *BveIF1A* is an important determinant of sodium tolerance (Rausell et al., 2003). Expression of *OseIF1* in *Oryza sativa* was up-regulated treatment with salt, ABA and mannitol; moreover, *OseIF1* plays a central role in salt stress adaptation in rice by regulating ion accumulation and the intracellular redox status (Diédhiou et al., 2008; Rangan et al., 2009). *eIF1* expression in *Porteresia coarctata* was also found to be induced by NaCl, ABA, and mannitol treatments (Latha et al., 2004). Regardless, few studies have focused on *eIF1A*-mediated abiotic stress regulation in woody plants. Accordingly, further investigation is needed to determine how physiological changes are controlled through eIF1A, such as the promotion of ROS scavenging systems to adjust to various environments.

*Tamarix hispida*, a shrub or small tree primarily distributed in saline or arid and semi-arid regions, is highly tolerant to salt, drought, and high temperature. Recently, a novel obligately halophilic, facultatively alkaliphilic actinobacterium, designated EGI 80759T, was isolated from the rhizosphere of *T. hispida* Willd, in Karamay, Xinjiang Province, northwestern China. Strain EGI 80759T exhibits obligately halophilic growth with tolerance to 8–25% (w/v) NaCl (optimum growth at 10–12%, w/v) and facultatively alkaliphilic growth within the pH range 7.0–11.0 (optimum growth at pH 9.0–10.0) (Zhang et al., 2016). Based on monitoring data of the groundwater level at typical sections in the lower reaches of Tarim River, the PSII photosynthetic activity of *T. hispida* under drought stress appears to decrease with increasing groundwater depth, and the increase in excess energy could result in a greater risk of photoinhibition. However, the good adaptability and drought tolerance of *T. hispida* could prevent serious PSII damage despite the presence of drought stress (Zhu et al., 2010). In addition, some abiotic stress response genes of *T. hispida* have been characterized, such as the V-type H^+^-ATPase c subunit (*ThVHAc1*) (Yang et al., 2016), WRKY transcript factor (Zheng et al., 2013), and *GST* (Yang et al., 2014). Such information makes *T. hispida* an ideal model for cloning stress tolerance genes and investigating the physiological and molecular mechanisms of stress responses in trees (Gao et al., 2008). In the current study, we cloned and characterized the *eIF1A* gene from *T. hispida* and found it to be strongly responsive to salt and osmotic stresses. *TheIF1A* improved salt and osmotic stress tolerance in transgenic plants by regulating stress-related gene expression, improving plant ROS scavenging ability, and decreasing cell damage and death. The results of these experiments provide evidence regarding the potential mechanism and physiological role of *eIF1A* in *T. hispida*.

## Results

### Cloning and Analysis of *TheIF1A* and its Promoter

The full-length *TheIF1A* gene is 432 bp and encodes a 143-aa protein with a predicted molecular weight of 16.40 kDa and a theoretical pI of 5.09. Multiple sequence alignment analysis indicated that the eIF1A proteins from different plants share a highly conserved eIF1A domain (Supplementary Figure S1A) located between residues 28 and 110 but without SANT, TUDOR, FN1, CNX, or PLDc features. Phylogenetic analysis indicates that TheIF1A is most similar to eIF1A from *B. vulgaris* (Supplementary Figure S1B).

A 1352-bp (from -1 to -1352) promoter region was cloned using thermal asymmetric interlaced polymerase chain reaction (TAIL-PCR). Searches using the PLACE algorithm demonstrated the presence of many *cis*-elements, such as ABRELATERD1, CAATBOX1, DOFCOREZM, MYB1AT, TATABOX2, and WRKY71OS, in this region, which indicates that *TheIF1A* may be regulated by different types of transcription factors (TFs) (Supplementary Figure S2).

### Expression of *TheIF1A* in *T. hispida*

**FIGURE 1:**
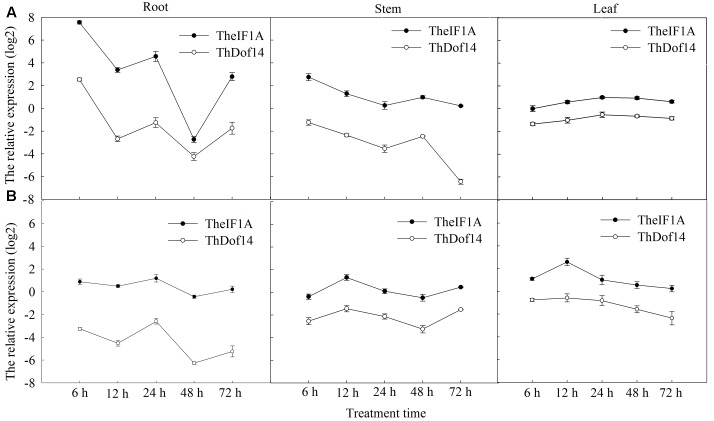
***TheIF1A* and *ThDof* expression patterns in different organs of *Tamarix hispida* in response to various treatments.** The relative expression level = transcription level under stress treatment/transcription level under control condition (0 h). And all the expression leves were log2 transformed. *β-Actin* (FJ618517), *α-tubulin* (FJ618518), and *β-tubulin* (FJ618519) were used as reference genes. Error bars were obtained from three replicates of real-time PCR, and every replicate included at least 20 seedlings used as biological replicates. **(A)** 0.4 M NaCl stress; **(B)** 20% (w/v) PEG_6000_ stress.

The results of qRT-PCR showed *TheIF1A* to be induced in response to NaCl and polyethylene glycol (PEG) treatments. After NaCl treatment for 6 h, expression of *TheIF1A* in roots was increased by 187.6-fold compared to the control. At 48 h, *TheIF1A* expression was down-regulated in roots compared to the control but up-regulated by 1.97-fold in stems (**Figure 1A**). Under PEG stress, *TheIF1A* transcription was considerably higher in leaves than in roots and stems, with *TheIF1A* transcripts in leaves being 4.22-fold higher in roots and 2.43-fold higher in stems at 12 h (**Figure 1B**).

**FIGURE 2:**
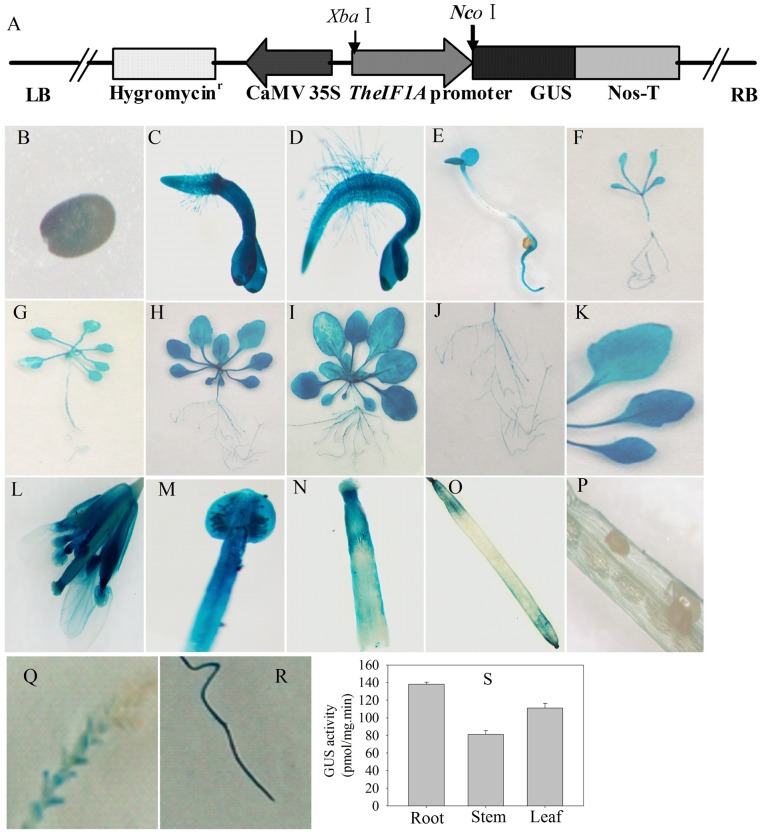
**Expression activity analysis of the *TheIF1A* promoter.** Transgenic *Arabidopsis* plants carrying the *TheIF1A* promoter and transiently expressing *T. hispida* seedlings were used. **(A)** Schematic map of the *TheIF1A* promoter inserted into the pCAMBIA1301 binary vector, which was used for *Arabidopsis* transformation. **(B–P)** Expression of *TheIF1A* at different growth stages and in different organs or tissues. **(B)** Mature seed; **(C)** 3-days-old seedling; **(D)** 5-days-old seedling; **(E)** 7-days-old seedling; **(F)** 10-days-old seedling; **(G)** 14-days-old-seedling; **(H)** 3-week-old seedling; **(I)** 5-week-old seedling; **(J,K)** root and three rosette leaves from a 3-week-old plant; **(L)** whole flower cluster; **(M)** bracteole; **(N)** stigma; **(O)** intact fresh silique; **(P)** dissected silique; **(Q)** stem and leaves of *T. hispida*; **(R)**, root of *T. hispida*; **(S)** GUS activity according to **(Q,S)**. Data represent the means ± SD of three independent experiments.

To further investigate expression via the *TheIF1A* promoter, transgenic *Arabidopsis* plants expressing *GUS* under the control of the *TheIF1A* promoter (namely, *pTheIF1A::GUS*) were generated (**Figure 2A**). The results showed GUS activity throughout the entire plant, except for in immature seeds of the silique (**Figures 2B–P**). Furthermore, expression levels differed among tissues and developmental stages. *pTheIF1A::GUS* was also transiently transformed into *T. hispida* seedlings followed by GUS staining, with the highest GUS activity in roots and the lowest in stems (**Figure 2Q–S**), further confirming the results for the tissue expression profile of *TheIF1A.*

### Analysis of Upstream Regulators of *TheIF1A*

**FIGURE 3:**
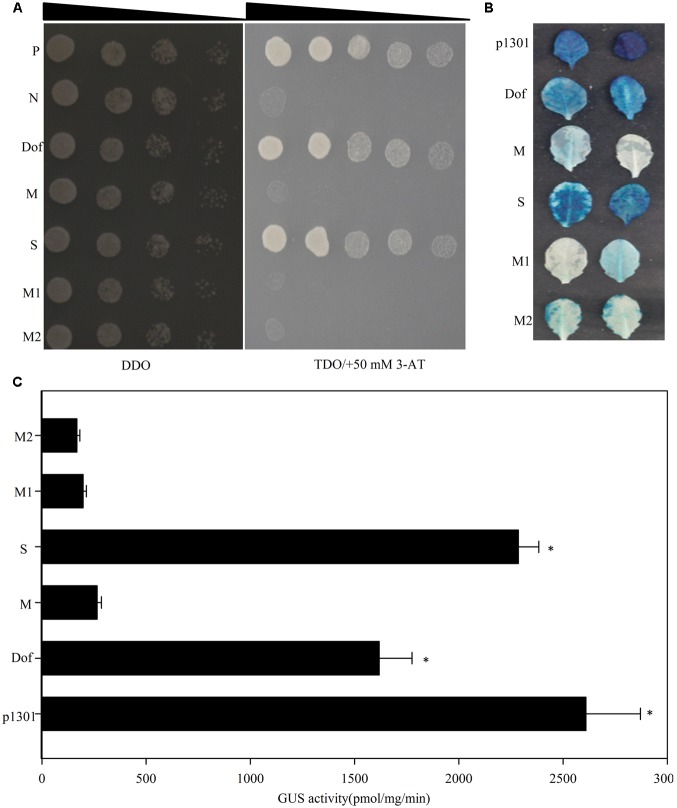
**Yeast one-hybrid analyses of upstream regulators of *TheIF1A*.** **(A)** P, positive control, the pGADT7-Rec2 vector encoding murine p53 fused with GAL4 AD. N, negative control, the pHIS2 reporter vector containing the *cis*-acting DNA consensus sequence recognized by p53. Dof, Dof motif. M, indicates a mutated Dof motif. S, The *TheIF1A* promoter fragment containing the Dof motif. M1, The *TheIF1A* promoter fragment not containing the Dof motif. M2, The *TheIF1A* promoter fragment containing the mutated Dof motif. Transformants spotted onto SD/-Leu/-Trp (DDO) were used as positive controls for transformant growth. Positive transformants were further confirmed by spotting serial dilutions (1/1, 1/10, 1/100, 1/1000, 1/10000) onto SD/-His/-Leu/-Trp plates with 50 mM 3-AT (TDO/+50 mM 3-AT); the triangle indicates the dilutions from 1 to 10000. **(B)** Results of transient reporter experiments for effector overexpression in *Arabidopsis*. p1301 and pCAMBIA1301 were used as positive controls. Dof, M, M1, and M2 were consistent with **(A)**. **(C)** GUS activity according to **(B)**. ^∗^indicates significant differences between the ‘M’ and other lines (*p* < 0.05).

Twelve Dof motifs were identified in the *TheIF1A* promoter, suggesting that *TheIF1A* is likely regulated by TFs that interact with this motif. A yeast one-hybrid assay was conducted to identify upstream regulators using the reporter vector pHIS2-*cis* (containing triple tandem Dof motif repeats) as bait to screen a *Tamarix* TF cDNA library. One protein (ThDof, KF896302) was found to bind to the Dof motif but failed to bind to mutant Dof motifs (**Figure 3A**). In addition, ThDof could bind to a truncated *TheIF1A* promoter retaining the Dof motif but could not bind to promoter fragments lacking the motif or with a mutated Dof motif (**Figure 3A**). These findings suggest that ThDof may specifically bind to the Dof motif and may regulate *TheIF1A* by binding to this motif in the *TheIF1A* promoter.

To validate these interactions, we co-transformed reporter plasmids (the motifs, promoter fragments and serially mutated motifs followed by a 46-bp minimal promoter were independently cloned into pCAMBIA1301) and the effector construct (pROKII-ThDof, Supplementary Figure S3B and **Figure 3B**) into *Arabidopsis* leaves. Measurement of GUS activity and histochemical staining both demonstrated that activation of GUS in *Arabidopsis* cells when co-transformed with reporters containing complete motifs or the *TheIF1A* promoter-including motifs. However, when the reporter contained a mutated motif or *TheIF1A* promoter fragments without a motif, the *Arabidopsis* cells displayed less GUS staining and decreased GUS activity (**Figures 3B,C**). These findings clearly confirmed that ThDof may activate expression of *TheIF1A* by binding to the Dof motif present in its promoter.

The expression patterns of *TheIF1A* and *ThDof* in *T. hispida* under salt and drought stresses were analyzed by qRT-PCR, and the results indicated that they share similar expression profiles. Under salt treatment, both genes exhibited peak transcription at 6 h in roots and stems, reaching peak levels in leaves at 24 h (**Figure 1A**). Under drought conditions, the lowest expression of both genes was observed at 48 h in roots and stems. In leaves, both genes showed highest and lowest expression at 12 and 72 h, respectively (**Figure 1B**).

### Overexpression of *TheIF1A* Improves Salt and Osmotic Stress Tolerance in Transgenic Tobacco

As *TheIF1A* was induced by NaCl and PEG stresses, we investigated whether overexpression of *TheIF1A* in plants enhances salt and osmosis tolerance using *35S::TheIF1A*-transgenic tobacco plants of the T_3_ generation. PCR analysis of the transgenic lines confirmed the successful transformation of *TheIF1A* into tobacco (Supplementary Figure S4A). The three lines of transgenic tobacco showing the highest expression were Lines 1, 3, and 9 (439.58-, 252.48-, and 508.46-fold compared with *tubulin*, respectively) (Supplementary Figure S4B), and these lines were selected for further analysis.

**FIGURE 4:**
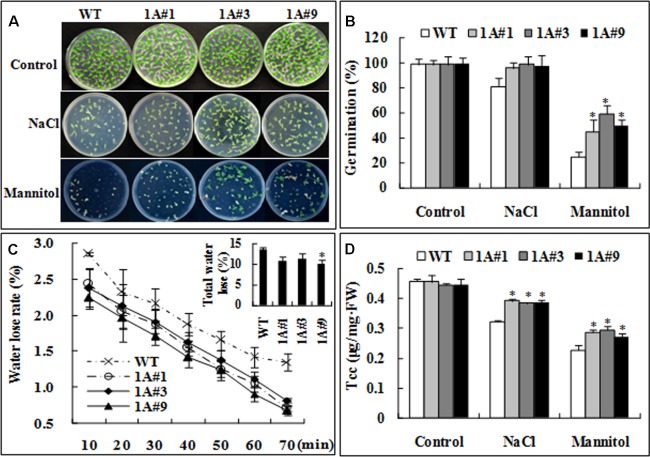
**Salt and osmotic stress tolerance analysis of WT and transgenic tobacco plants.** WT, Wild type; 1A#1, 1A#3, and 1A#9 are three transgenic *TheIF1A* lines. **(A)** Germination assay under NaCl and mannitol treatment for 12 days. **(B)** Germination percentage according to **(A)**. **(C)** The weight of water loss from 28-days-old of WT and *TheIF1A* transgenic plants in 70 min. **(D)** Total chlorophyll was compared after 6 days of NaCl or mannitol treatment of 28-days-old of WT and *TheIF1A* transgenic plants. The experiments were repeated at least three times, and 30 tobacco seedlings were used for each treatment. ^∗^indicates significant differences between transgenic lines and WT (*p* < 0.05).

Germination rates were compared between the transgenic lines and wild type (WT) plants. Without treatment, there was no observable difference between WT and transgenic plants (**Figure 4A**). However, germination in the transgenic plants was significantly increased compared with WT under mannitol stress (**Figures 4A,B**) (*p* < 0.05). The water loss rates of the transgenic and WT plants (**Figure 4C**) were compared after leaves were exposed to air for 10 min, and the rate of water loss in the transgenic lines was 83% of that in WT; after 70 min, the water loss rate of the transgenic lines was also significantly decreased (80%) compared with WT plants. Although the chlorophyll content (tcc) of the transgenic plants was different from that in WT plants prior to salt or mannitol stress, tcc in all plants decreased strongly after 6 days of salt or mannitol stress. Nonetheless, the transgenic lines showed significantly higher tcc than WT plants (the difference was, on average, 1.20–1.26-fold) (**Figure 4D**), which suggests that overexpression of *TheIF1A* prevents chlorophyll loss under salt or mannitol stress conditions.

**FIGURE 5:**
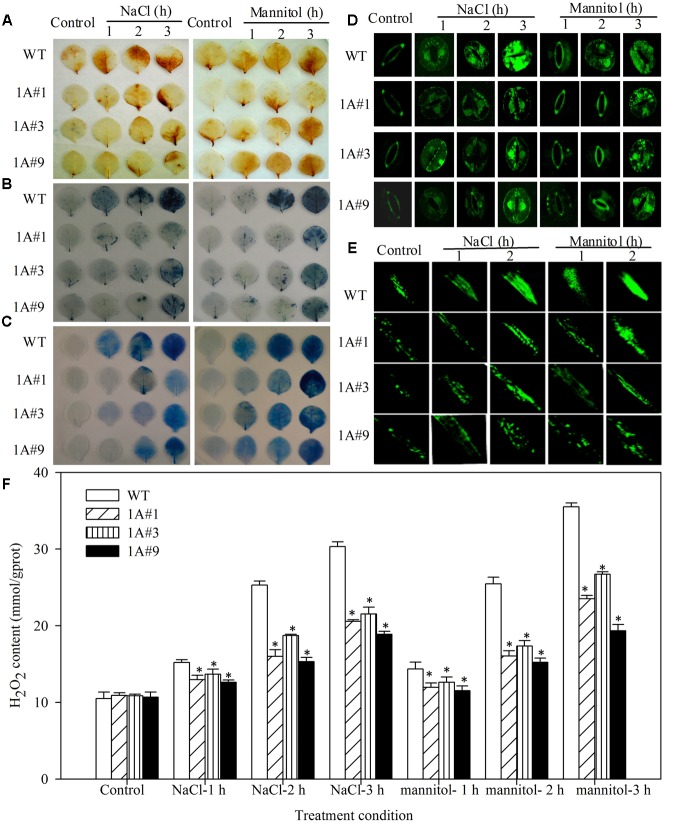
**Reactive oxygen species (ROS) levels and cell death in *TheIF1A* transgenic tobacco plants and WT under different stress conditions.** All experiments were repeated at least three times, and approximately 15 leaves collected from multiple 28-days-old seedlings were inspected in each experiment. **(A)** DAB staining indicates accumulation of H_2_O_2_ in leaves of WT and transgenic plants subjected to 1, 2, or 3 h of salt or mannitol stress. **(B)** NBT staining of O^2-^ accumulation in leaves according to DAB staining. **(C)** Evans blue staining. **(D)** Representative microscopy images of ROS production in intact guard cells, as indicated by the fluorescent dye DCF. Epidermal peels were loaded with H_2_DCF-DA for 10 min after incubation in fixing buffer for 2 h. **(E)** ROS production in the root, as indicated by the fluorescent dye DCF. Main roots detached from control, NaCl and mannitol stress (1 h) plants were incubated in incubation buffer for 2 h at room temperature and subsequently stained with 5 μM H_2_DCF-DA for 10 min. **(F)** The total H_2_O_2_ content according to the staining **(A–F)**. ^∗^indicates significant differences between transgenic lines and WT (*p* < 0.05).

To understand the regulation of physiological responses, physiological parameters involved in stresses were compared, including the ROS content, the activity of protective enzymes, such as SOD, POD, GST and GPX, EL rates and the MDA content. Staining with diaminobenzidine (DAB) and nitroblue tetrazolium (NBT) demonstrated that H_2_O_2_ and O^2-^ levels, respectively, in leaves were similar in WT and transgenic lines under no treatment. However, WT plants exhibited increased H_2_O_2_ and O^2-^ accumulation compared with transgenic lines, particularly at 1 h after stress treatment, at which point the degree of staining in the transgenic lines was notably decreased compared with the WT plants (**Figures 5A,B**). ROS levels in intact guard cells and the main roots, as determined by stained with H_2_DCF, were similar to the levels indicated by DAB and NBT staining. That is, with the exception of 0 h, WT plants exhibited increased ROS accumulation in both guard cells and main roots compared with transgenic lines (**Figures 5D,E**). Additionally, the H_2_O_2_ content according to the degree of staining showed significantly less H_2_O_2_ in *TheIF1A*-transgenic plants than in WT plants (**Figure 5F**, *p* < 0.05). These findings indicate that *TheIF1A* overexpression leads to a significant decrease in ROS accumulation in plant cells under salt and mannitol stress conditions.

**FIGURE 6:**
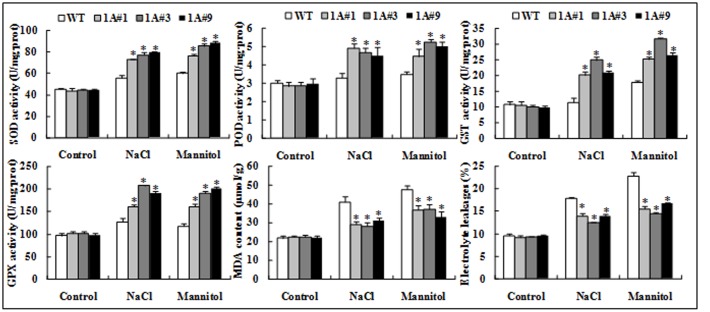
**Physiological index analyses of 28-days-old seedlings of *TheIF1A* transgenic plants and WT under 100 mM NaCl or 200 mM mannitol for 6 days; well-watered seedlings were used as the control.** All experiments were repeated three times. Data represent the means ± SD of three independent experiments. ^∗^ Indicates significant differences between the transgenic lines and WT (*p* < 0.05).

Superoxide dismutase, POD, GST, and GPX are key ROS scavengers, and our results demonstrated similar changes in the activities of these enzymes. Under no treatment, no significant differences in the activities of these enzymes were observed between transgenic and WT plants. However, the activities of these enzymes in the transgenic plants were significantly (*p* < 0.05) increased compared with the WT plants under salt and mannitol stresses. For example, the SOD activity of the transgenic lines was, on average, approximately 1.4-fold that of WT under both stresses, and POD activity in the former was 1.41-fold that of the latter under salt stress. GST activity of 1A#3 was 2.16-fold that of WT exposed to NaCl, and GPX activity of 1A#3 was 1.63- and 1.62-fold of that of WT in response to NaCl and mannitol, respectively. Although the MDA content did not differ between transgenic lines and WT plants under no stress, the transgenic plants accumulated significantly less MDA compared with the WT plants after treatment. The average MDA content of WT was 1.40- and 1.23-fold of the transgenic lines under NaCl and mannitol stresses, respectively (**Figure 6**). These findings indicated that overexpression of *TheIF1A* increased enzyme activities, subsequently decreasing MDA accumulation.

In addition, the plasma membrane system was analyzed using Evans blue staining and EL rate determination. The tested lines did not exhibit apparent differences under control conditions. However, when treated with salt or mannitol, the transgenic plants exhibited weaker Evans blue staining and a lower EL rate compared with WT plants (the EL of WT was, on average, 1.33-fold higher than that of transgenic lines exposed to NaCl and 1.47-fold following mannitol), and these differences were significant (**Figures 5C**, **6**).

Taken together, these findings suggested that *TheIF1A* overexpression improves salt and osmotic stress tolerance by increasing ROS scavenging and preventing cell damage to maintain growth.

### Expression Analysis of Stress-related Genes in *TheIF1A*-transformed Tobacco Plants

**FIGURE 7:**
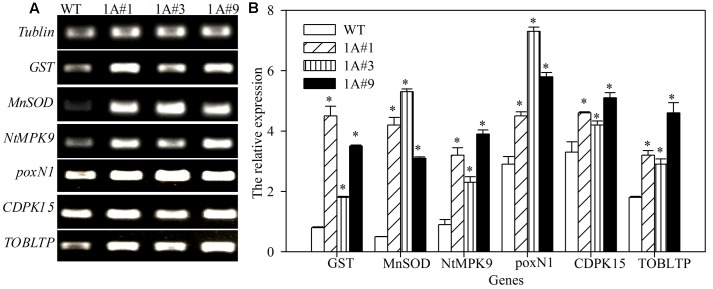
**Reverse-transcription-polymerase chain reaction (RT-PCR) analyses of stress-related genes in *TheIF1A*-transformed tobacco plants. (A)** Gel electrophoresis of tobacco *GST, MnSOD, poxN1, NtMPK9, CDPK15*, and *TOBLTP*. *Tubulin* was used as the reference gene. **(B)** Relative expression level according to *tubulin* based on **(A)**. All experiments were repeated three times. Data represent the means ± SD of three independent experiments. ^∗^ Indicates significant differences between the transgenic lines and WT (*p* < 0.05).

Tobacco plants overexpressing *TheIF1A* showed resistance to NaCl and mannitol stresses, and the activities of the corresponding protective enzymes were increased in transgenic lines compared with WT plants, suggesting that expression of key potential stress-related genes may be altered by exogenous expression of *TheIF1A*. Stress response-related genes were selected for analysis, and seven tobacco genes, including *GST, MnSOD, NtMPK9, poxN1, CDPK15*, and *TOBLTP*, exhibited increased levels of expression in *TheIF1A*-overexpressing lines compared with WT plants. In particular, levels of *GST* and *MnSOD* expression were 5.63- and 10.60-fold higher, respectively, than those in WT plants (**Figure 7**). These findings indicated that *TheIF1A* improves ROS scavenging via regulation of antioxidant genes.

### Transient Expression Analysis of *TheIF1A* in *T. hispida*

**FIGURE 8:**
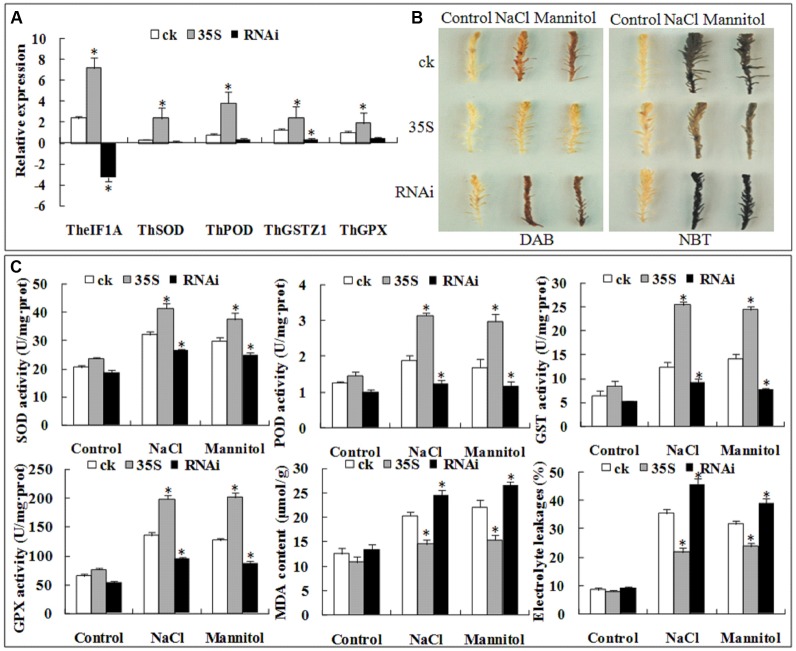
**Transient expression analyses of *TheIF1A* in *T. hispida* under 100 mM NaCl or 200 mM mannitol treatments for 1 h. ck, 35S, and RNAi indicate the empty pROKII, *35S::TheIF1A*, and RNAi::*TheIF1A*-transformed lines, respectively. (A)** Expression of *TheIF1A*, *ThSOD*, *ThPOD*, *ThGSTZ1*, and *ThGPX* in transient expression lines according to the reference gene. **(B)** DAB and NBT staining. **(C)** SOD, POD, GST, and GPX activities, the MDA content and EL of transiently expressing *T. hispida* seedlings. All experiments were repeated three times. Data represent the means ± SD of three independent experiments. ^∗^ Indicates significant differences between pROKII and *35S::TheIF1A* and between pROKII and *RNAi::TheIF1A* lines (*p* < 0.05).

To further confirm the role of *TheIF1A* in the *T. hispida* response to abiotic stress, *35S::TheIF1A* and *RNAi::TheIF1A* were transiently transformed into *T. hispida*, and *TheIF1A* expression in two lines was determined by qRT-PCR. The results showed the highest *TheIF1A* expression in the *35S::TheIF1A* plants and the lowest in the *RNAi::TheIF1A* plants. The level of *TheIF1A* expression in the overexpression line was 27.9-fold higher than that in the control plants and was 50.01-fold that in the *RNAi::TheIF1A* plants under no stress. These findings indicate the successful overexpression of *TheIF1A* in the *35S::TheIF1A* plants and the *RNAi-*mediated silencing of *TheIF1A* in the *RNAi::TheIF1A* plants (**Figure 8A**). Interestingly, the expression patterns of *ThSOD*, *ThPOD*, *ThGSTZ1*, and *ThGPX* were similar to that of *TheIF1A*. Expression of these genes was highest in *35S::TheIF1A* lines and lowest in *RNAi::TheIF1A* lines (**Figure 8A**), which suggests that the abiotic stress response of *TheIF1A* may correlate with the level of *ThSOD*, *ThPOD*, *ThGSTZ1*, and *ThGPX* expression.

The related physiological indexes were determined. Under NaCl treatment, SOD, POD, GST, and GPX activities in the control plants were 49∼77% of the activities in the *35S::TheIF1A* plants, and the activities of these enzymes in the *RNAi::TheIF1A* plants were 36∼63% of the activities in the *35S::TheIF1A* lines. After mannitol treatment, SOD, POD, GST, and GPX activities in the control plants were 56∼77% of the activities in the *35S::TheIF1A* plants; the activities of these enzymes in the *RNAi::TheIF1A* lines were 32∼67% of the activities in the *35S::TheIF1A* seedlings. The MDA content in the *35S::TheIF1A* plants was 71% of that in the control plants and 59% of that in the *RNAi::TheIF1A* plants under NaCl stress. After treatment with mannitol, the MDA content in the *35S::TheIF1A* plants was 70% of that in the control plants and 58% of that in the *RNAi::TheIF1A* lines. The EL rate in the *35S::TheIF1A* plants was 61% of that in the control plants and 48% of that in the *RNAi::TheIF1A* lines under NaCl stress. However, after exposure to mannitol, the EL rate in the *35S::TheIF1A* plants was 75% of that in the control plants and 61% of that in the *RNAi::TheIF1A* plants (**Figure 8C**), which indicates that *TheIF1A* effectively increased enzymatic activity and decreased cell damage.

Furthermore, DAB, NBT and Evans blue staining also demonstrated that *35S::TheIF1A T. hispida* seedlings accumulated less ROS and suffered less cell damage and that *RNAi::TheIF1A* plants accumulated more ROS and suffered more cell damage compared with control plants after treatment with 100 mM NaCl or 200 mM mannitol (**Figure 8B**). Taken together, these findings indicate that *TheIF1A* improved plant tolerance via regulation of other stress-related genes, the management of ROS homeostasis, and the prevention of cell damage and death.

## Discussion

Some reports have indicated the involvement of e*IF1A* in the response to abiotic stress. For example, sugar beet *BveIF1A* not only partially complemented a yeast eIF1A-deficient strain but also increased the NaCl tolerance of *BveIF1A* transgenic *Arabidopsis* plants (Rausell et al., 2003). The assembly, kinetics and composition of stress granules in yeast may also be related to (eIF)3, eIF4A/B, eIF5B, and eIF1A proteins (Buchan et al., 2011). Expression of *LceIF1* from *Leymus chinensis* (Trin.) was decreased under sodium-saline stresses, whereas, overexpression was induced under sodic-alkaline stresses; however, when expressed in most organisms under no treatment, *eIF1*-transgenic lines exhibited relatively high *eIF1* expression, which resulted in potentially increased stress resistance (Sun and Hong, 2013). These findings indicate that *eIF1(A)* may also play a positive role in response to abiotic stress tolerance. Thus, the *eIF1A* gene from the halophyte *T. hispida* warrants investigation under exposure to abiotic stress. Here, we characterized *TheIF1A* in plants exposed to salt and drought, and our results provide a new and further perspective on the function of *eIF1(A)* in stress responses.

In the present study, *TheIF1A* expression in *T. hispida* was induced in roots, stems, and leaves after treatment of plants with NaCl and PEG. In a previous study, Diédhiou et al. (2008) demonstrated that *eIF1* transcription was only increased in salt-tolerant plants after prolonged salt treatment, whereas, the expression levels in salt-sensitive plants were not induced. The *eIF1* gene from the salt-tolerant plant *Porteresia coarctata* was only induced after treatment with NaCl for 3 and 5 days (Latha et al., 2004). In addition, *BveIF1A* was not induced under NaCl stress for 24 h, whereas, *AteIF1A* expression was slightly decreased under the same stress (Rausell et al., 2003). Our results indicate that *TheIF1A* is induced by NaCl and PEG stress and that *TheIF1A* may play a potential role in salt and PEG tolerance.

The results of *TheIF1A* promoter GUS staining showed that *TheIF1A* was expressed throughout all plant parts at every developmental stage, with the exception of fresh siliques and seeds. Transient transformation of the *TheIF1A* promoter into *T. hispida* seedlings also resulted in different tissue expression patterns. Conversely, Wang et al. (2012) indicated that *TaeIF5A1* exhibited similar expression patterns in different tissues and growth stages, which suggests that *TaeIF5A1* may function in stress tolerance via gene regulation in all tissues. *TaeIF3g* was induced in leaves under drought stress, and *TaeIF3g* overexpression improved plant drought tolerance (Singh et al., 2007). Several other *eIF*s are also reportedly regulated in response to different abiotic stresses (Costa-Neto et al., 2006; Wang et al., 2012). However, there are few reports on the role of *eIF1A* in abiotic stress responses based on physiological regulation, such as tcc, protective enzymes, and ROS levels.

Accordingly, *TheIF1A* was overexpressed in tobacco to further understand the involvement of this gene in stress resistance. Germination, hydration, and chlorophyll production are important for normal plant growth, and these processes are disrupted during stress. Specifically, the water content, chlorophyll level and net photosynthetic rate are vital for plant development. The tcc level of *TaeIF5A-* or *ThWRKY4-*overexpressing *Arabidopsis* was increased compared with that of WT plants, suggesting that *TaeIF5A* and *ThWRKY4* regulate salt tolerance genes (Wang et al., 2012; Zheng et al., 2013). In the present study, *TheIF1A* was associated with these indicators, and tobacco plants overexpressing *TheIF1A* exhibited increased germination rates, decreased water loss rates and more stable tcc compared with WT and control plants under salt or mannitol stresses; these results suggest that *TheIF1A* may participate in physiological regulation in plants. Furthermore, the potential ROS metabolism system was examined to verify the regulation mechanism of *TheIF1A*, and the results indicated that *TheIF1A* overexpression positively enhanced the activities of plant protective enzymes and reduced ROS accumulation under NaCl or mannitol stresses. Taken together, the current findings suggest that *TheIF1A* expression improves the activity of antioxidant enzymes, thereby enhancing ROS scavenging.

Previous studies have indicated that compared with WT plants, *MnSOD* overexpression effectively improved SOD activity and salt stress tolerance (Tanaka et al., 1999). Plant *POD* genes are involved in several different physiological functions, including growth regulation and wound healing; many *poxN* genes are wound inducible and display low induction under no treatment, whereas, expression of these genes is rapidly induced after exposure to abiotic stress (Ito et al., 2000). GSTs are a superfamily of multifunctional, dimeric enzymes that participate in detoxification of endo- and xenobiotics, and some *GST* genes have been implicated in responses to dehydration (Kiyosue et al., 1993; Bianchi et al., 2002), hydrogen peroxide (H_2_O_2_) and salicylic acid (SA) signaling (Chen et al., 1996), herbicide application (Edwards et al., 2000), wounding (Vollenweider et al., 2000), auxin production (Chen and Singh, 2002), and salt stress (Moons, 2003). In a previous study, we demonstrated that *ThGSTZ1* effectively improves plant resistance to salt and mannitol stresses (Yang et al., 2014). MPK is a member of the MKK2 pathway, which mediates cold and salt stress signaling in *Arabidopsis* (Teige et al., 2004). Popescu et al. (2009) demonstrated that MPK phosphorylation enriched TFs involved in regulating development, defense, and stress in plants. As osmotic stress induces calcium signaling, *CDPK* (calcium-dependent protein kinase) genes are prime candidates as the link between calcium signaling and downstream responses (Zhu, 2002). CDPK activates a stress-inducible promoter, which bypasses the stress signals that have various functions mediated through different CDPKs, suggesting that these factors act as potential positive regulators that control stress signal transduction in plants (Sheen, 1996). *TOBLTP* encodes a lipid transfer protein, the expression of which is triggered by ABA and drought (Torres-Schumann et al., 1992; Treviño and O’Connell, 1998). Considering that the activity of several protective enzymes is enhanced, it is important to determine the expression profiles of these genes in *TheIF1A*-overexpressing plants. The induced expression of *GST, MnSOD, NtMPK9, poxN1, CDPK15*, and *TOBLTP* in *TheIF1A*-transgenic tobacco plants suggests that *TheIF1A* may be involved in the regulation of these genes to enhance abiotic stress tolerance. Furthermore, transient expression of *TheIF1A* in *T. hispida* seedlings increased expression of *ThSOD*, *ThPOD*, *ThGSTZ1*, and *ThGPX* in *35S::TheIF1A* plants, whereas it reduced expression of these genes in *RNAi::TheIF1A* lines. Moreover, all plants exhibited increased expression levels under NaCl or mannitol treatment. These findings suggest that all of these genes are responsive to stress, providing candidate regulators in stress regulation pathways. The results of these analyses suggested that *TheIF1A* regulates other genes, such as SOD and POD, to enhance related activities, increase ROS scavenging and decrease ROS accumulation.

Many motifs, such as DOFCOREZM, MYBCORE, and WRKY71OS, were identified in the promoter of *TheIF1A*, a finding that suggests the role of *TheIF1A* in the response to abiotic stress involves different TFs. Dof TFs are important positive regulators in plant responses to adverse stimuli. Tomato *DOF* genes exhibit distinct diurnal expression patterns and are differentially induced in response to osmotic, salt, heat, and low-temperature stresses. *SlCDF1-* or *SlCDF3*-transgenic *Arabidopsis* plants exhibit increased drought and salt tolerance (Corrales et al., 2014). The expression profiles of nine *BraDof* family genes under cold, heat, salt, and drought treatments in ‘*Lubaisanhao*’ and ‘*Qingdao 87-114*’ were examined by qRT-PCR, and most were up-regulated (Ma et al., 2015). Thus, we predict that *TheIF1A* may respond to salt and drought stresses in a manner similar to that of *Dof* genes. Zheng et al. (2013) reported that *ThDof* is an upstream regulator of *ThWRKY4* in the regulation of *ThWRKY4* expression and function. Dof may also interact with and stimulate the DNA binding of stress-responsive bZIP proteins (Kang and Singh, 2000). Many Dof domain proteins enhance growth and tolerance to dehydration and salt stress (Foyer and Noctor, 2005; Shukla et al., 2006). Based on these previous studies and the current results obtained from yeast one-hybrid assays and expression analyses, we speculate that *ThDof* is an upstream regulator of *TheIF1A* that may regulate induction of the latter by binding to the Dof motif in its promoter and that these two proteins may function in the same stress regulation pathway.

## Conclusion

**FIGURE 9:**
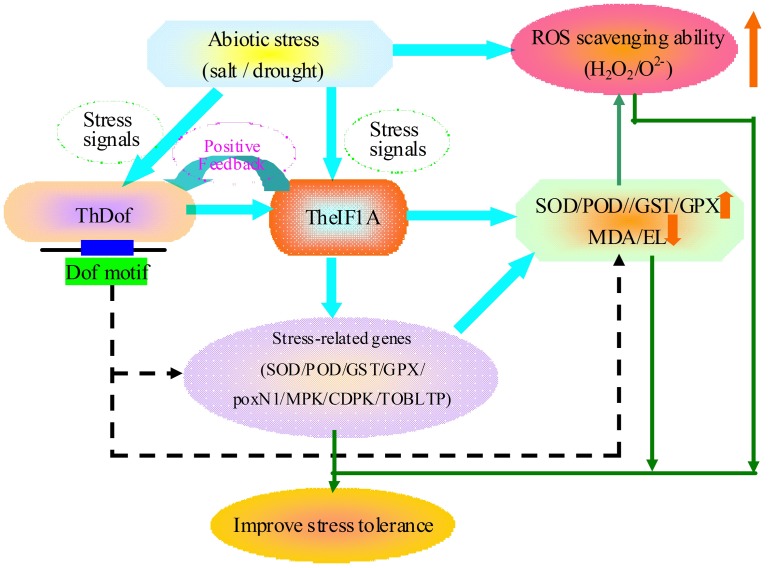
**Mapping of the potential tolerance regulation mechanism of *TheIF1A*.** The solid line with arrows is based on our results, whereas, the dotted line with arrows is speculation based on the current and previous studies.

We demonstrated that *TheIF1A* acts as a stress response regulator to improve plant salt and osmosis tolerance via the maintenance of tcc and by increasing the activity of protective enzymes, decreasing ROS generation and regulating other related genes to improve the ROS scavenging ability and reduce cell damage (**Figure 9**). Furthermore, ThDof specially binds to the Dof motif in the *TheIF1A* promoter and may regulate or interact with TheIF1A to participate in salt and osmotic stress responses in plants.

## Materials and Methods

### Plant Materials and Treatments

*Tamarix hispida* and *Arabidopsis thaliana* (Col, WT) seedlings were cultivated in pots containing a mixture of turf peat and sand (2:1 v/v) in a greenhouse for 2 months under 14 h light/10 h dark, 70–75% relative humidity, an average temperature of 24°C and well-watered conditions. Two-month-old *T. hispida* seedlings were exposed to the following treatments: 400 mM NaCl or 20% (w/v) PEG_6000_ for 6, 12, 24, 48, or 72 h. The roots, stems, and leaves from at least 20 seedlings were then independently harvested at the indicated time points after each treatment and pooled for qRT-PCR analyses. Seedlings irrigated with water alone were used as the control; every treatment was carried out three times as biological replicates, with every replicate containing at least 20 seedlings.

### Cloning of the *TheIF1A* Gene and its Promoter

The *TheIF1A* gene (KF801668) was cloned from the *T. hispida* leaf cDNA library (Gao et al., 2008) The phylogenetic tree of eIF1A proteins from different plant species was constructed using the Neighbor-Joining method (Saitou and Nei, 1987). The *TheIF1A* promoter was PCR amplified from *T. hispida* genomic DNA using a genome walking kit. *cis*-elements in the *TheIF1A* promoter were identified using the PLACE database^1^ (Higo et al., 1999). T_3_ seedlings of *TheIF1A* promoter transgenic line were used for temporal and spatial expression analyses of *TheIF1A* via GUS staining (Clough and Bent, 1998). Moreover, pCAMBIA1301*-TheIF1A* was transiently expressed in *T. hispida* seedlings to verify the *TheIF1A* expression results obtained in *A. thaliana*.

### RNA Isolation and qRT-PCR

Total RNA was isolated from seedlings exposed to different treatments using the CTAB method (Wang et al., 2002); 0.5 μg of RNA was used for reverse transcription with PrimeScript^TM^ RT reagent Kit (Takara). The resulting cDNA product was diluted 10-fold and used for RT-PCR analyses. qRT-PCR was applied using an MJ Opticon^TM2^ machine (Bio-Rad, Hercules, CA, USA); the reaction system and procedures were performed according to Gao et al. (2011). *β-Actin* (FJ618517), *α-tubulin* (FJ618518), and *β-tubulin* (FJ618519) were used as reference genes. The primer sequences are listed in Supplementary Table S1. Relative expression levels were calculated using the delta-delta Ct method (Livak and Schmittgen, 2001). The RT-PCR data for the transgenic tobacco lines were normalized to *tubulin* (AJ421412) (Ban et al., 2011). Three independent experiments were performed, and at least 20 seedlings were used in each treatment.

### Identification of the Upstream Regulator of *TheIF1A*

Twelve Dof motifs (core base sequence “AAAG”) were identified in the *TheIF1A* promoter (Supplementary Figure S2). A yeast one-hybrid assay (Clontech, Palo Alto, CA, USA) was used to investigate specific genes recognizing these motifs and activating *TheIF1A* expression. The three tandem copies of the promoter fragment containing the Dof motif core (“AAAAGT”) sequence were cloned into the pHIS2 vector (Supplementary Figures S3A,B). TFs from different families were identified, PCR amplified and subsequently cloned into pGADT7-Rec2 (Clontech) to produce a cDNA library used in the one-hybrid assay (Zheng et al., 2013).

To determine interactions between the Dof motif and related positive clones, a mutated Dof core motif “AAAG” with “CCCT” (Dof-M) was constructed. Furthermore, fragments of the *TheIF1A* promoter containing the Dof motif (Dof-S), excluding the Dof motif (Dof-S-M1) or containing a mutated Dof motif (Dof-S-M2) were cloned into pHIS2 (Supplementary Figure S3B). Cells transformed with the empty p53HIS2 plasmid (pHIS2 contains three copies of the p53 DNA element) were used as a negative control in the yeast one-hybrid assay. All primers are presented in Supplementary Table S2.

To confirm these interactions, all constructs were fused with a *CaMV35S*-46 minimal promoter and cloned into pCAMBIA1301 for expression of the *GUS* gene. The full-length cDNA of *ThDof* was cloned into pROKII under the control of the 35S promoter (referred to as pROKII-ThDof) to create effecter vectors (Supplementary Figure S3C), which were transformed into *Arabidopsis* using the floral dip method (Clough and Bent, 1998). A T_3_ generation line of *ThDof-*transgenic *Arabidopsis* was then used for transient expression of all Dof reporters using *Agrobacterium*-mediated transformation. All co-transformed *Arabidopsis* leaves were stained to measure GUS activity (Jefferson et al., 1987; Jefferson, 1989). Furthermore, the expression patterns of *TheIF1A* and *ThDof* under salt and drought conditions in *T. hispida* were analyzed by qRT-PCR.

### Generation of Transgenic Tobacco

The full-length cDNA of *TheIF1A* was amplified and cloned into pROKII (referred to as *35S::TheIF1A*). The primer sequences are presented in Supplementary Table S2. *35S::TheIF1A* was transferred into tobacco (*Nicotiana tabacum*) using *Agrobacterium*-mediated transformation. Sixty-one lines were generated in the T_0_ generation, which were further selected until the T_3_ generation; 12 different lines were detected by PCR, and the expression levels were analyzed by qRT-PCR. Three transgenic lines (Lines 1, 3, and 9, referred to as 1A#1, 1A#3, and 1A#9, respectively) with the highest expression levels were selected for further analysis.

### Stress Tolerance Analysis

For the seed germination assay, seeds from WT and transgenic lines were sown onto 1/2 MS agar medium containing either 100 mM NaCl or 200 mM mannitol. Germination was recorded after 12 days using germination rate = germinated plantlets number/total seeds sown^∗^100%. For stress tolerance assays, 28-days-old WT and transgenic plants were treated with 100 mM NaCl or 200 mM mannitol for 6 days. Leaves were used to determine tcc, SOD activity, POD activity, GPX activity, GST activity and the MDA content using a corresponding commercially available kit from Nanjing Jiancheng Bioengineering Institute (Nanjing, China). SOD activity was assessed based on the auto-oxidation of hydroxylamine. POD activity was tested using a peroxidase assay kit, with absorbance at 340 and 420 nm. GST activity was determined using a glutathione *S*-transferase (GSH-ST) assay kit (Colorimetric method). GPX (GSH-Px) activity was determined by the velocity method using a GSH-Px kit; the reaction was initiated by the addition of H_2_O_2_, and the change in absorbance during the conversion of GSH to GSSG was recorded spectrophotometrically at 412 nm. The MDA content was determined based on thiobarbituric acid (TBA) reactivity; the developed red color of the resulting reaction was measured at 532 nm using a spectrophotometer. The H_2_O_2_ content was assessed on the basis of a hydrogen peroxide assay kit (Minotti et al., 1994; Kwak et al., 2005; Ban et al., 2011; Liu et al., 2013). The EL rate was evaluated per the report of Yang et al. (2014). For water-holding ability, the aerial parts of seedlings were placed onto clean filter paper and dried for 70 min. The fresh weights of the samples were measured every 10 min to determine the rate of water loss relative to each time interval.

In addition, leaves from seedlings treated with 100 mM NaCl or 200 mM mannitol for 0 (control), 1, 2, and 3 h were detached and immediately used for histochemical staining analysis. O^2-^ accumulation, H_2_O_2_ accumulation, and cell death in the leaves were assayed through NBT, DAB, and Evans blue staining, respectively. ROS production in intact guard cells and roots was assessed by staining with 5 μM H_2_DCF-DA (Fluka) (Fryer et al., 2002; Zhang et al., 2011). ROS accumulation was visualized using an LSM410 confocal laser scanning microscope (CLSM) (Zeiss, Jena, Germany) with excitation at 488 nm and an emission at 525 nm, and images were acquired using ZEN 2009 light edition imaging software (Zhang et al., 2011). The corresponding H_2_O_2_ content under the same treatments as staining assays was also examined. Furthermore, stress-related genes (*TOBLTP*, *GST*, *MnSOD*, *NtMPK9*, *poxN1*, and *CDPK15*) in *TheIF1A-*overexpressing tobacco plants were investigated by RT-PCR using the primers presented in Supplementary Table S3. All experiments were performed three times.

### Transient Expression of *TheIF1A* in *T. hispida*

To better analyze the salt and mannitol response of *TheIF1A*, 295-bp of the *TheIF1A* sequence was cloned into a reconstructive ProKII vector to construct the RNAi-suppression vector *RNAi::TheIF1A* (Supplementary Figure S5) (Zhang et al., 2012; Li et al., 2014). The forward and reverse primers are presented in Supplementary Table S2. A single colony of EHA105 harboring *35S::TheIF1A*, *RNAi::TheIF1A* and the empty ProKII vector (used as a control, labeled ck) was cultivated at 28°C to OD_600_ = 0.6, after which the cells were harvested by centrifugation and adjusted to OD_600_ = 0.2 using 1/2 MS solution (pH = 5.8) containing 3% (w/v) sucrose, as the transformation solution supplied with 150 μM acetosyringone. Two-month-old *T. hispida* seedlings were soaked in the transformation solution for 48 h at 25°C and 30–40 rpm, and the culture solution was changed every 4 h (Zheng et al., 2012). The transformed seedlings were treated with 100 mM NaCl or 200 mM mannitol for 1 h. DAB, NBT and Evans blue staining was subsequently used to visualize the O^2-^ and H_2_O_2_ contents and cell damage in the leaves; POD, SOD, GST, GPX activities and the MDA content were also examined. In addition, the expression levels of stress-related genes and *ThSOD*, *ThPOD*, *ThGSTZ1*, and *ThGPX* in seedlings of these three transgenic lines were examined by qRT-PCR. The primers are presented in Supplementary Table S1. All experiments were performed three times. Every independent replicate included at least 12 seedlings per line.

### Statistical Analysis

All data were analyzed using Statistical Package for Social Science (SPSS) (SPSS, Chicago, IL, USA). Differences among the tested lines were calculated using Tukey’s multiple comparison tests. The significance level was set as *p* < 0.05, and the standard deviation (SD) was calculated to analyze sample variability.

## Author Contributions

GY wrote the manuscript and performed some of the assays (data shown in **Figures 2**, **3**, **5**, **8**, **9**). LY performed most of the assays (obtained the transgenic tobacco plants and cloned the promoter; data shown in **Figures 1**, **4**, **6**, **7**). YW and CW performed the data analysis and also revised the manuscript. CG provided funds for the current study, designed the study and revised the manuscript.

## Conflict of Interest Statement

The authors declare that the research was conducted in the absence of any commercial or financial relationships that could be construed as a potential conflict of interest.

## Footnotes

1 http://bioinformatics.psb.ugent.be/webtools/plantcare/html/

## Abbreviations

ABA: abscisic acid
COR: cold responsive
DAB: 3,3′-diaminobenzidine tetrahydrochloride
EL: electrolyte leakage
GPX: glutathione peroxidase
GST: glutathione transferase
H_2_DCF-DA, 2: 7-dichlorofluorescein diacetate
MDA: malondialdehyde
MW: molecular weight
pI: isoelectric point
POD: peroxidase
qRT-PCR: quantitative real-time polymerase chain reaction
ROS: reactive oxygen species
SOD: superoxide dismutase
tcc: total chlorophyll content.
