# Supplementary material for: The Translation Initiation Factor 1A (TheIF1A) from Tamarix hispida Is Regulated by a Dof Transcription Factor and Increased Abiotic Stress Tolerance
Source: Front Plant Sci. 2017 Apr 7;8:513. doi: 10.3389/fpls.2017.00513 (PMC5383729; doi:10.3389/fpls.2017.00513)

Title page

The translation initiation factor 1A (*TheIF1A*) from *Tamarix hispida* is regulated by a Dof transcription factor and increased abiotic stress tolerance

Guiyan Yang, Lili Yu, Yucheng Wang, Chao Wang, Caiqiu Gao*

State Key Laboratory of Tree Genetics and Breeding, Northeast Forestry University, Harbin, China

*Corresponding author: Caiqiu Gao

E-mail: gaocaiqiu@yahoo.com

Tel: +86-451-82191820

Fax: +86-451-82191822

Supplementary tables: Primers used in the study.

Table S1 The primer sequences used in real-time RT-PCR

Gene	Primer sequence
TheIF1A	5’- AAGGGAGGCAAGAACAGG -3’
	5’- ACCAGCAGCAATCCAGAC -3’
ThDof	5’- GACTGGCTGCAGGGTACGAT -3’
	5’- GAGAGGCTGTAGTTGTTGTAG -3’
ThSOD	5’- AGCTGTGGCAATTATCGC -3’
	5’- CAATGATGTTACCCAGGT -3’
ThPOD	5’- CACCACCAGAAGAACAAATCG -3’
	5’- CCCTAATGAAACAGTCATGG -3’
ThGSTZ1	5’- GCCTGCACTTGGACTTAA -3’
	5’- TCACGCTCTGTATCACCC -3’
ThGPX	5’- CCAATTCTATCTACGATT -3’
	5’- TCCTGGCTCTTGCCAAGC-3’
Actin	5’- AAACAATGGCTGATGCTG -3’
	5’- ACAATACCGTGCTCAATAGG -3’
α-tubulin	5’- CACCCACCGTTGTTCCAG -3’
	5’- ACCGTCGTCATCTTCACC -3’
β-tubulin	5’- GGAAGCCATAGAAAGACC -3’
	5’- CAACAAATGTGGGATGCT -3’

Table S2 The primer sequences used in one-hybrid assay and reporter and effector plasmids

construct	Forward Primers (5’-3’)	Reverse Primers (5’-3’)
pHIS2-Dof	5’- AATTCAAAAGTAAAAGTAAAAGTGAGCT-3’	5’- *C*ACTTTTACTTTTACTTTT*G*-3’
pHIS2-Dof-M	5’- AATTCACCCTTACCCTTACCCTTGAGCT-3’	5’- CACCCTTACCCTTACCCTTG-3’
pHIS2-Dof-S	5’- GGAATTCCAAAAGTTTAGCCTTAACACT-3’	5’- AATTTAAGTTAACTTATACTAGAGCTCG-3’
pHIS2-Dof-S-M1	5’- GGAATTCCATTTAGCCTTAACACTTAGT-3’	5’- AATTTAAGTTAACTTATACTAGAGCTCG-3’
pHIS2-Dof-S-M2	5’- GGAATTCCACCCTTTTAGCCTTAACACT-3’	5’- AATTTAAGTTAACTTATACTAGAGCTCG-3’
pCAM-Dof	5’-AGCTTAAAAGTAAAAGTAAAAGTCCCTTCCTCTAT<br>ATAAGGAAGTTCATTTCATTTGGAGAGAACACGGC-3’	5’-CATGGCCGTGTTCTCTCCAAATGAAATGAACTTCCT<br>TATATAGAGGAAGGGACTTTTACTTTTACTTTTA
pCAM-Dof-M	5’-AGCTTACCCTTACCCTTACCCTTCCCTTCCTCTATA<br>TAAGGAAGTTCATTTCATTTGGAGAGAACACGGC-3’	5’-CATGGCCGTGTTCTCTCCAAATGAAATGAACTTCCT<br>TATATAGAGGAAGGGACCCTTACCCTTACCCTTA
pCAM-Dof-S	5’- AGCTTCAAAAGTTTAGCCTTAACACTC-3’	5’-CATGGCCGTGTTCTCTCCAAATGAAATGAACTTCCT<br>TATATAGAGGAAGGGAATTTAAGTTAACTTATACTAA
pCAM-Dof-S-M1	AGCTTCATTTAGCCTTAACACTTAGTC-3’	5’-CATGGCCGTGTTCTCTCCAAATGAAATGAACTTCCT<br>TATATAGAGGAAGGGAATTTAAGTTAACTTATACTAA
pCAM-Dof-S-M2	5’- AGCTTCACCCTTTTAGCCTTAACACTC-3’	5’-CATGGCCGTGTTCTCTCCAAATGAAATGAACTTCCT<br>TATATAGAGGAAGGGAATTTAAGTTAACTTATACTAA
pROKII-ThDof	5’- ATCGTCTAGAATGAGCGAGGATGGAGGATT	5’- CGATGGTACCTTAAACCAAGGGATTTGTTG
pROKII-TheIF1A	5’-ATCGGGTACCATGCCGAAGAACAAGGGGAAGG -3’	5’- CGATGGTACCTTAAATCTTGTCAATATCCTCG -3’

Table S3 The primer sequences used in RT-PCR

Gene	GeneBank number	Primer sequence
tublin	AJ421412	5’-TAACCATCATAGAAGAGGGTTC -3’<br>5’-GCAATCCTTCTTGACAATGAGG -3’
TOBLTP	D13952	5’- GTAAGGAATGTTGACACCAC -3’<br>5’- GAATGGAAATGGTAGGTAAG -3’
GST	D10524	5’- TTGGCCTTCTACTTCCATCC -3’<br>5’- TGTCAACTGCAACCATGAGAG -3’
MnSOD	AB093097	5’- GCAACTCCACGGCTTCCAGACC -3’<br>5’- CGATAGCCCAACCAAGAGAACC -3’
NtMPK9	JX076823	5’- ATGTGCTGCTATCGACAC -3’<br>5’- ATCAGATTCCATAAGCTC -3’
poxN1	AB027753	5’- ATCGGTCCACAGCTTCTGGTCTG -3’<br>5’- AGGGTCTCAACACTCAAGATC -3’
CDPK15	JN662020	5’- CTGCTTCAATGTGTAGAG -3’<br>5’- TAGTAAGCACTCCCAACT -3’

Fig. S1 Multiple-sequence alignment and Phylogenetic tree of the deduced amino acid sequence of *TheIF1A* protein with the *eIF1A* proteins from other species. (A) Multiple sequence alignment. Identical amino acids are highlighted with a black background. (B) Phylogenetic tree of the *TheIF1A* and some *eIF1A* based on sequence alignments of the encoded proteins.

A

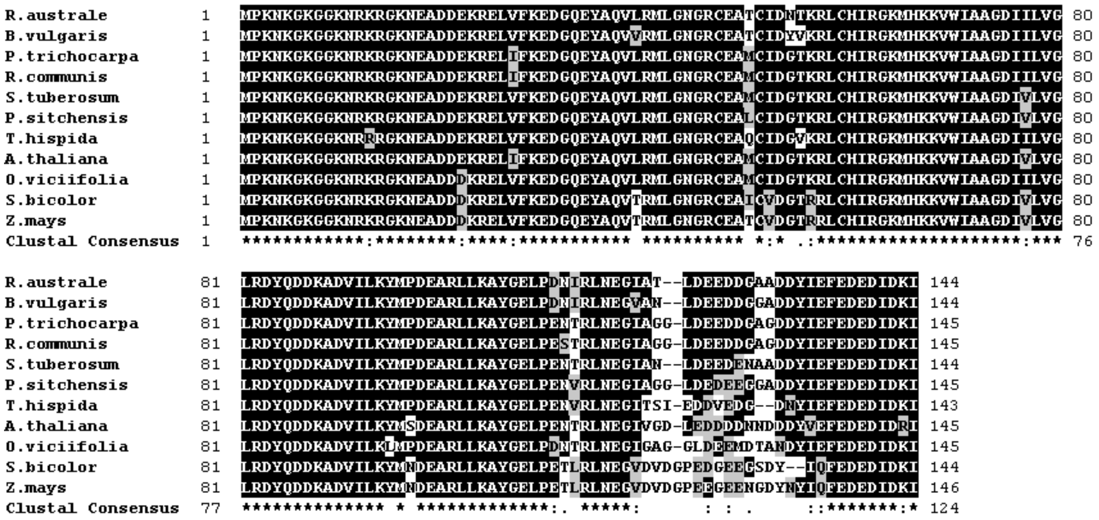

B

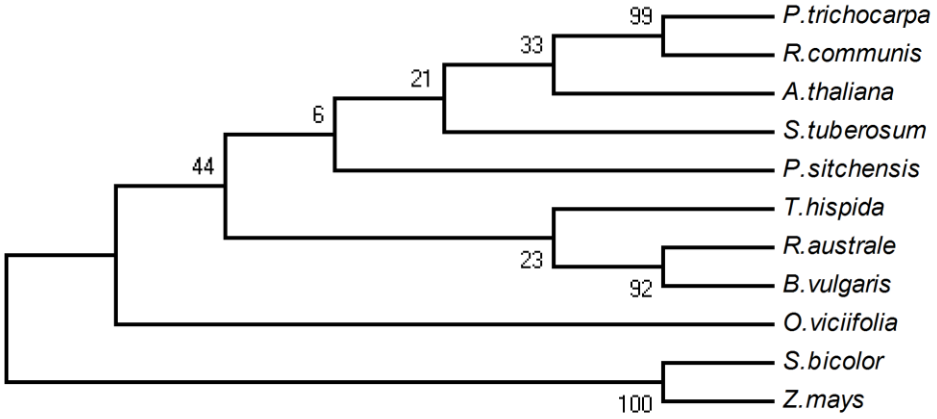

Fig. S2 Mapping the cis-elements in the TheIF1A promter

ACTTCCAGATGAATGGGCATGAGTAGTATA*TGAC*CTTTAGTGGTATGTGTGTTATATGAA*TG*
WRKY710S WRKY710S

*AC*TATCGTATTT*CAAT*TAATATATGCATAATTAAATGTTC*ACGT*ATTTTTATGTGTTCTATGCG
CAATBOX1 ACGTATERD1

AACCTTGCATATTAGTTTTGTATAATAGGTTACT*CAAT*ACTGTAGTACTAA*TGAC*TTAATGAG
CAATBOX1 WRKY710S

TTTCATTTCCTGGTTCCCTACCTATT*CAAT*GGGTAGTTTTTACTTTGTAAGAATACTTTGAGGG
CAATBOX1

TAAGGTTTGATGGCTTCTTATATGGGTCGAGAGTTAAGGTTGTCCTAGGGTTCGTCTAAC***AT***
NODCON1GM

CTTT*TGAC*CCAGTCGTTCTAAAATCTTATTATTTTATGTAATTTTATTTT***CACT****AGATT*TTATAA
WRKY710S CACTFTPPCA1 ARR1AT

CCACAAA*CAGTTG*GTTTACTT*AGATT*GATGGTTAAGTTATT*TGAC*TCCCTTTT*CACT*AATATTT
MYBCORE ARR1AT WRKY710S CACTFTPPCA1

TAATTGTATTACTAATTTAAGTAGACA***AAAG***TTTAGCCTTAA*CACT*TAGTATAAGAATAGTA
DOFCORECR CACTFTPPCA1

GCGGGTGCCAGACTTATGCTAGGTGTGGTGGTAATGGCA*CACT*ATTTTGTAACTAATGCTTC
CACTFTPPCA1

CTCAAACAAATATTTTAAGTATTAAATTTGGAAGTGTTACA*ATAGAA*TTGATTATTG*AGATT*A
BOXIINTPATPB ARR1AT

GCATATTTATTATAG*CAAT*ATATGGTATTTAAAAATATTTGGGATTGCAAATCATCTTATTTA
CAATBOX1

GTATAAGTTAACTTAAATTTTTTGAGGTAAATATATT*AATAAA*TGA*GATA*TTTATATCTAACTA
POLASIG1 GATABOX

AGTAAAACTATACTACAAGA*TAACAAA****ATGGATA***AAT*AAAG*CACC*CGTCA*T*TGAC*T*AAAG*CTA
AMYBBOX1 AMYBBOX2 DOFCORECR ASF1MOTIFCAMV WRKY710S DOFCORECR

CCGGTTGGGAAGTTGGCTTTTTGTAGG*CACT*TGTTGTCTCAAAC*ACGT*ATTTCTTATAGCCAT
ACGTATERD1

GAAACATATTCAG*CAAT*TTACCTCTATCGTGTTGGCTTA*TGAC*AGGGTTGAAGACATTGGCTA
CAATBOX1 WRKY710S

A*GATA*AGAGTATTGAATTTTTGA*AATAAA*TAAAA*TATAAAT*AATTAAT*GATA*AAATATAATTAT
GATABOX POLASIG1 TATABOX GATABOX

TGAGCTATTACCTGCATTTTTTTATAATGGGGATCCAACTTTACT*GATA*TAACCT*ATAGAA*TCT
GATABOX BOXIINTPATPB

AC*CAAT*CGCTTGAGTCAGCTATCAAGCCGCCTGTAAAAATACCGTAGTCATATTAAAATTCC
CAATBOX1

ATAACGATCTGGTAGCTGCAAGTCTGCAAGCCTGCAACGCCATCTATAAAACCCTCCACGCG

AGCGAAG*AAAG*AGAGGCGGTAAGCGGTCGCAAGAAGCGACACAAATCCTGAAAACC*CAAT*C
DOFCORECR

GCCGCTAAACTAAG*CATTTG*A*CACT*TCGTTGTTGCGGAAACAACCTATAGATCTAGGGTTTTG
CAATBOX1 MYCCONSENSUSAT CACTFTPPCA1

TAGAGAGAAG*AAAG*AGAGTAGAACCGAAG ATG
DOFCORECR

Fig. S3 (A) Scheme of the Dof motif and the fragments of *TheIF1A* promoter containing the Dof motif in the whole *TheIF1A* promoter. (B) Scheme of reporter and effector vectors. In the reporter, the black rectangle indicates the cis-acting element Dof, and the white rectangle indicates the mutant. Δpromoter indicates the promoter fragment of *TheIF1A* that contains the Dof motif, and Δm-promoter indicates the promoter fragment that contains the mutated Dof motif. In the effector, gene means *ThDof14*. (C) Scheme of reporter and effector used in the transient co-expression of Arabidopsis.

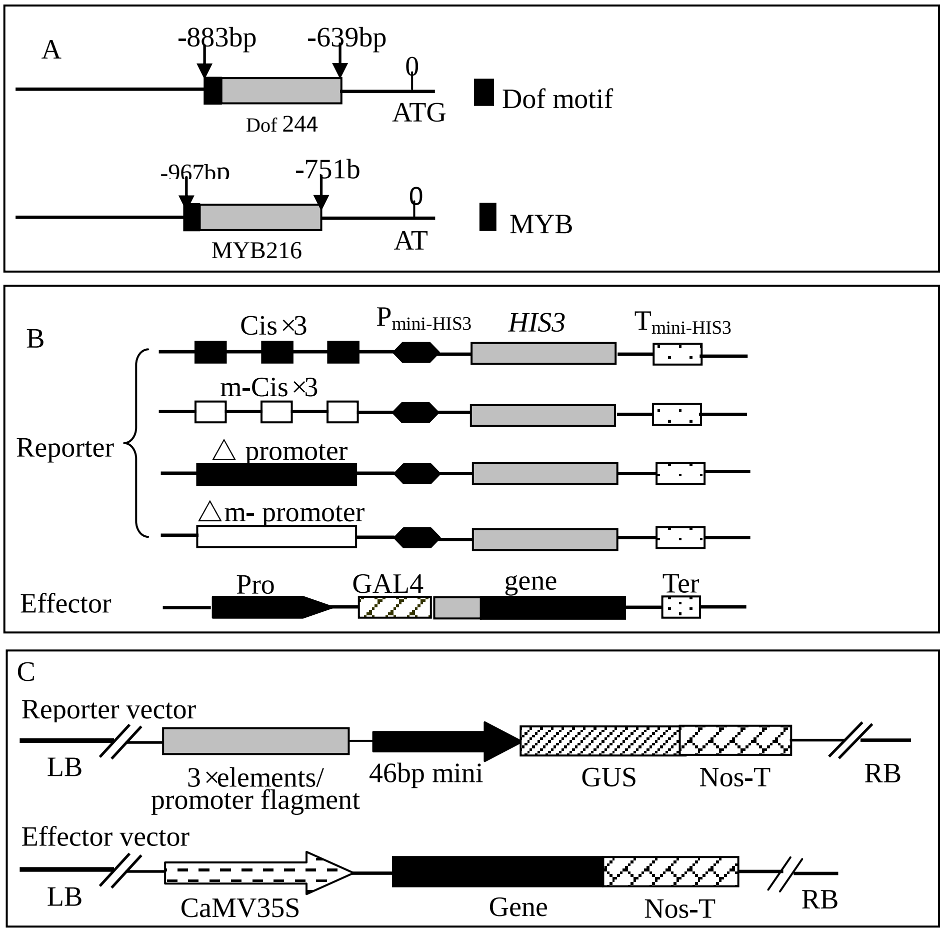

Fig. S4 The expression analysis of TheIF1A in transgenic lines. (A) PCR analysis of transgenic lines. M, marker, 1. WT, 2, positive control. 3-14, 12 transgenic lines. (B) qRT-PCR analysis of the 12 transgenic lines.

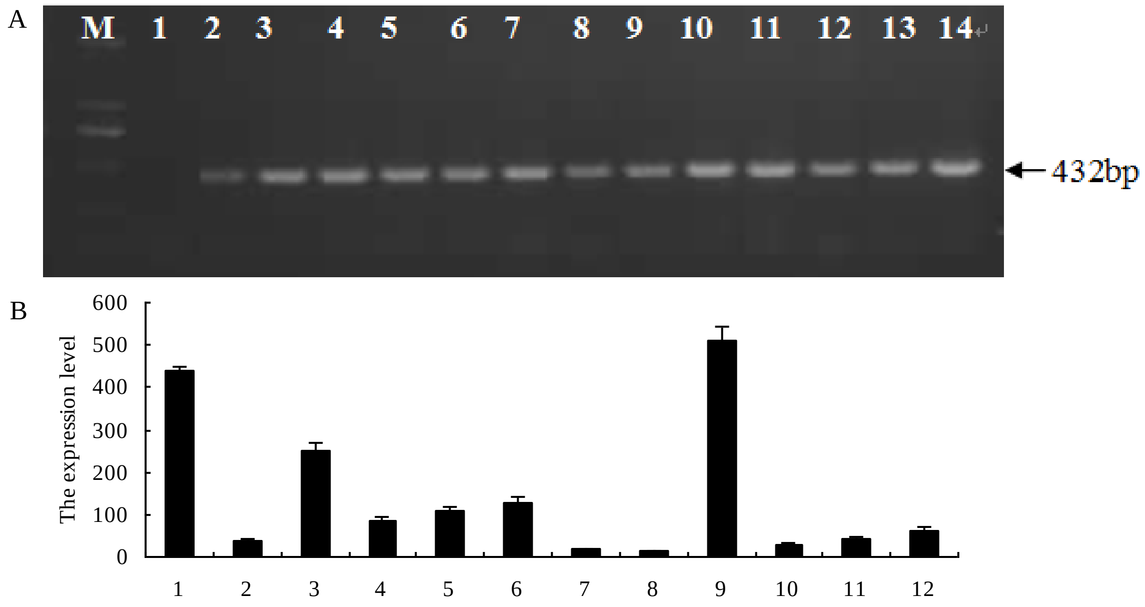

Fig. S5 The reconstructive ProKII vector (*RNAi::TheIF1A*)

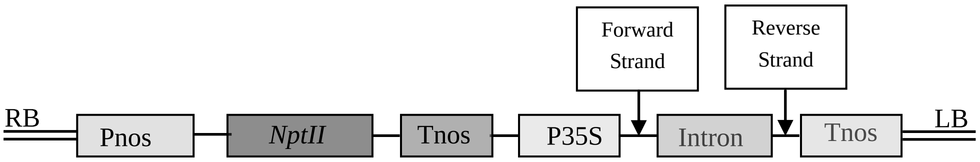

Supplement: Supplementary file 1 [file Data_Sheet_1.pdf]
